# A novel chemotherapeutic sensitivity-testing system based on collagen gel droplet embedded 3D–culture methods for hepatocellular carcinoma

**DOI:** 10.1186/s12885-017-3706-6

**Published:** 2017-11-08

**Authors:** Jun Hou, Zhixian Hong, Fan Feng, Yantao Chai, Yunkai Zhang, Qiyu Jiang, Yan Hu, Shunquan Wu, Yingsong Wu, Xunian Gao, Qiong Chen, Yong Wan, Jingfeng Bi, Zheng Zhang

**Affiliations:** 1 Research Center for Clinical and Translational Medicine, the 302nd Hospital of Chinese PLA, Beijing, 100039 People’s Republic of China; 2 Department of Hepatobiliary Surgery, the 302nd Hospital of Chinese PLA, Beijing, 100039 People’s Republic of China; 3 School of Laboratory Medicine and Biotechnology, Southern Medical University, Guangzhou, 510515 People’s Republic of China; 4 Research Institute, Guangzhou Darui Biotechnology Co Ltd, Guangzhou, 510515 People’s Republic of China

**Keywords:** Hepatocellular carcinoma, Multi-drug resistance, Collagen gel droplet embedded 3D–culture system, Chemotherapies

## Abstract

**Background:**

Patients suffering from advanced stage hepatocellular carcinoma (HCC) often exhibit a poor prognosis or dismal clinical outcomes due to ineffective chemotherapy or a multi-drug resistance (MDR) process. Thus, it is urgent to develop a new chemotherapeutic sensitivity testing system for HCC treatment. The presence study investigated the potential application of a novel chemotherapeutic sensitivity-testing system based on a collagen gel droplet embedded 3D–culture system (CD-DST).

**Methods:**

Primary cells were separating from surgical resection specimens and then tested by CD-DST. To identify whether HCC cell lines or cells separating from clinical specimens contain MDR features, the cells were treated with an *IC*_*50*_ (half maximal inhibitory concentration) or *IC*_*max*_ (maximal inhibitory concentration) concentration of antitumor agents, e.g., 5-furuolouracil (5-FU), paclitaxel (PAC), cisplatin (CDDP), epirubicin (EPI), or oxaliplatin (L-OHP), and the inhibitory rates (IRs) were calculated.

**Results:**

HepG2 cells were sensitive to 5-FU, PAC, CDDP, EPI, or L-OHP; the *IC*_*50*_ value is 0.83 ± 0.45 μg/ml, 0.03 ± 0.02 μg/ml, 1.15 ± 0.75 μg/ml, 0.09 ± 0.03 μg/ml, or 1.76 ± 0.44 μg/ml, respectively. Only eight (8/26), nine (9/26), or five (5/26) patients were sensitive to the *IC*_*max*_ concentration of CDDP, EPI, or L-OHP; whereas only three (3/26), four (4/26), or two (2/26) patients were sensitive to the *IC*_*50*_ concentration of CDDP, EPI, or L-OHP. No patients were sensitive to 5-FU or PAC.

**Conclusions:**

The in vitro drug sensitivity exanimation revealed the MDR features of HCC and examined the sensitivity of HCC cells from clinical specimens to anti-tumor agents. CD-DST may be a useful method to predict the potential clinical benefits of anticancer agents for HCC patients.

## Background

HCC is currently one of the most common causes of cancer-related deaths in China and the Asian-Pacific region [1–3]. In China, there are 466,100 new cases, and 422,100 deaths occur per year [2–5]. Although hepatic resection may be the first choice of treatment, only a small proportion (10–15%) of patients are suitable [6, 7]. Patients often suffer from advanced stage HCC upon their initial diagnosis and exhibit poor prognosis due to multi-drug resistance (MDR) features [8–10]. Thus, it is urgent and significant to develop a new chemotherapeutic sensitivity testing system for HCC treatment. Previously, the sensitivity of HCC cells to anti-tumor agents is often tested via MTT (3-(4, 5-dimethyl-2-thiazolyl)-2, 5-diphenyl-2-H-tetrazolium bromide) assays or SRB (Sulforhodamine B) assays based on the two-dimensional (2-D) monolayer culture [11]. However, cancer cells in solid tumors grow in a three-dimensional (3-D) pattern [12]. In this respect, an in vitro 2-D cell cultural model is limited in evaluating the clinical efficacy of chemotherapies [13]. Therefore, it is urgent to establish an in vitro model reflecting the 3-D culture of tumor cells.

CD-DST is a mature test in which primary-culture cells are embedded in collagen droplets [11–16]. Applications of CD-DST in chemotherapeutic sensitivity examination have been demonstrated in human cancers, e.g., lung cancer, gastric cancer, breast cancer, or colorectal cancer [11–16]. However, reports focused on potential applications and efficacy of CD-DST in HCC remain rare [17, 18]. This work aims to identify potential applications of CD-DST in HCC.

## Methods

### Cell lines and reagents

HepG2 (an HCC cell line) cells (Cat. 3111C0001CCC000035) or HepG2/ADR (an HCC cell line resistant to Adriamycin), which were purchased from Cell Culture Center, Institute of Basic Medical Sciences, Chinese Academy of Medical Sciences, or descripted previously [8], were cultured in complete DMEM (Invitrogen, Carlsbad, CA, USA) in a sterile incubator maintained at 37 °C, 5% CO_2_. Anti-tumor agents, 5-furuolouracil (5-FU; Beijing Union Pharmaceutical Factory, China), Paclitaxel (PAC; Shanghai XudongHaipu Pharmaceutical CO., LTD., China), Cisplatin (CDDP; HaosohPharma, China), Epirubicin (EPI; Pfizer, USA), and Oxaliplatin (L-OHP; JiangshuHengrui Medicine CO, LTD, China) were purchased.

### Human tissue samples

HCC specimens were obtained from surgical resections and preserved by our lab (from June 2015 and February 2016.). All patients with HBV (Hepatitis B Virus) - or HCV (Hepatitis C Virus) -related HCC, diagnosed based on an imaging examination confirmed by needle core biopsy, were included. Tumor stages were classified following the Barcelona Clinic Liver Cancer (BCLC) staging classification system [19, 20]. The histological grades of HCC were identified based on Edmondson’s grading system: well-differentiated tumors, grade I or I–II; moderately differentiated tumors, grade II or II–III; and poorly differentiated umors, grade III or III–IV. Table 1 showed the clinical features of 26 patients.

**Table 1:** Baseline characteristics of patients

Clinical features	Values (number (%))
Age(yr)	51.3 ± 7.4
Gender	
Male	20 (76.92%)
Female	6 (23.08%)
Aetiology	
Hbs-Ag positive	23 (88.46%)
HCV-Ab positive	3 (11.54%)
Child-Pugh score	
Class A	26 (100%)
Class B	0 (0%)
Tumour size	
< 3 cm	19 (73.07%)
3-5 cm	7 (26.93%)
Tumour number	
Single	16 (61.53%)
2–3	11 (38.47%)
BCLC staging	
Stage A	18 (69.23%)
Stage B	8 (30.77%)
Tumour differentiation	
well	9 (34.61%)
moderate	10 (38.46%)
poorly	7 (26.92%)
AFP	545 ± 892

### CD-DST

collagen gel droplet embedded 3D–culture system (CD-DST) was performed using a Kit Tumor Chemosensitivity Assay (Collagen gel Droplet culture method) provided by Guangzhou Darui Biotechnology Co., Ltd. (Guangzhou, China), according to the manufacturer’s instructions. For primary-culture cells of HCC, 0.2–0.5 g fresh-clinical specimens were digested by trypsin and incubated in collagen gel-coated flasks after eliminating blood cells or dead cells. Next, cells were collected and incubated in a collagen gel droplet embedded culture at 2 × 10^5^ cells/ml of collagen gel droplet (30 μl per drop). Then, cells were treated with the indicted concentration of anticancer agents (Table 2) for 24 h. After treatment with the anticancer agents, cells were incubated in a serum-free medium (4 ml PCM-2 medium to inhibit fibroblast proliferation) for 5–7 days to form colonies. Living cells were stained with neutral red, fixed with neutral formalin buffer, and directly examined/quantified by the cultured cells analysis system DR6690 (Guangzhou Darui Biotechnology Co., Ltd., China). The baseline (0-time group) of cells was indicated as the blank group. Inhibitory rates (IRs) were calculated as (O.D. 540 nm control group − O.D. 540 nm administration group) / (O.D. 540 nm control group − O.D. 540 nm blank group) × 100%. Relative survival rates = 100% - IR. For the cell line test (HepG2 cells), cells were harvested and analyzed using CD-DST tests. An IR value >25% indicated that cells were sensitive to the compound.

**Table 2:** Concentration of the anticancer drugs

Compounds	Concentration				
	μg/ml				
5-PU	0.10	0.50	1.00	2.00	5.00
PAC	0.01	0.02	0.05	0.08	0.10
CDDP	0.10	0.20	0.50	1.00	2.00
EPI	0.01	0.05	0.10	0.50	1.00
L-OHP	0.10	0..50	1.00	2.00	5.00

### Statistical analyses

Data are expressed as the mean ± SD. Statistical analyses were performed using the paired t-test or Fisher’s exact probability test. The *IC*_*50*_ and *IC*_*max*_ values were calculated using Origin 8.5 software.

## Results

### HepG2 cell cultures in collagen gel droplets

**Fig. 1:**
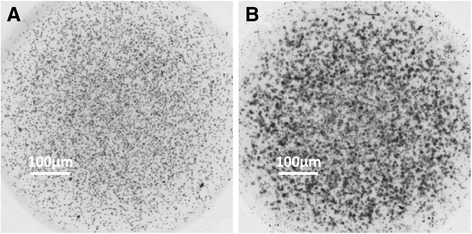
HepG2 cell cultures in collagen gel droplets. HepG2 cells were seeded in collagen gel droplets and analyzed by a multifunctional microplate reader at 540 nm. **a** The represent grey scale photograph of baseline (0-time) HepG2 cells; (**b**) The represent grey scale photograph of HepG2 cells after 5–7 days culturing

To establish the CD-DST methods, HepG2 cells were cultured in collagen gel droplets. The *O.D.* 540 nm values were examined at indicated time-points from three independent experiments. As shown in Table 3, similar results were obtained from each experiment, and the growth rates of HepG2 were from 4.1 to 4.62-fold (Table 3). These results were further confirmed by representative photographs. As shown in Fig. 1, after 5–7 days’ growth, the colony sizes were much larger than that at baseline.

**Table 3:** Growth rates of HepG2 cell in collagen gel droplets

Test Batch	Baseline (0-time)	Solvent Control (6 days)	Growth rate (GR)
	*O.D.* 540 nm		
1	22.18	97.95	4.1
	25.45	92.33	
	22.11	95.47	
2	26.29	108.51	4.56
	23.48	95.29	
	18.21	106.31	
3	19.83	111.03	4.62
	23.21	114.15	
	29.61	110.76

### HepG2 cells are sensitive to anti-tumor agents

**Fig. 2:**
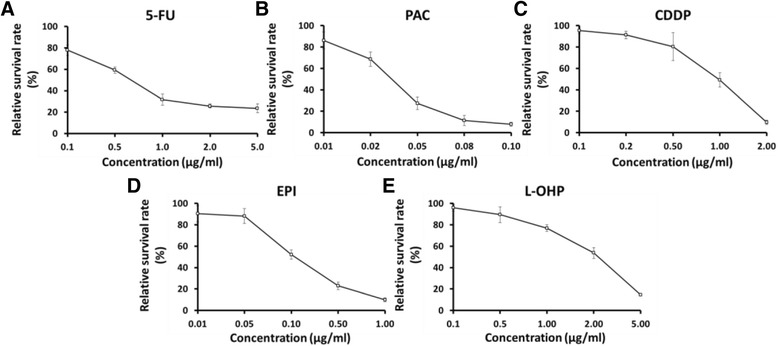
Effects of anti-tumor agents on HepG2 cells. **a-e** HepG2 cells were treated with indicted concentration of 5-FU, PAC, CDDP, EPI or L-OHP and analyzed by CD-DST. The results are shown as mean ± SD from three experiments with similar results

**Fig. 3:**
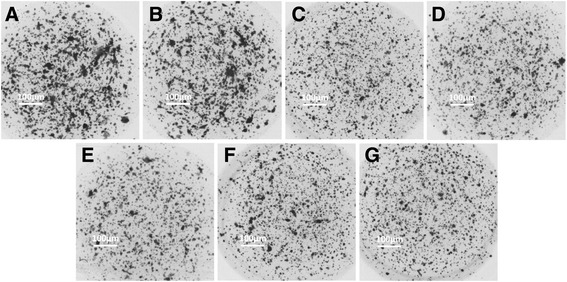
The represent figures of *IC*_*50*_ concentration anti-tumor agents on 3-D cultured HepG2 cells. **a** HepG2 cells were cultured in a 3-D manner. **b-g** HepG2 cells were treated with solvent control (**b**), or *IC*_*50*_ concentration of agents, 0.8 μg/ml 5-FU (**c**), 0.3 μg/ml PAC (**d**), 1 μg/ml CDDP (**e**), 0.9 μg/ml EPI (**f**) or 1.7 μg/ml L-OHP (**g**)

**Fig. 4:**
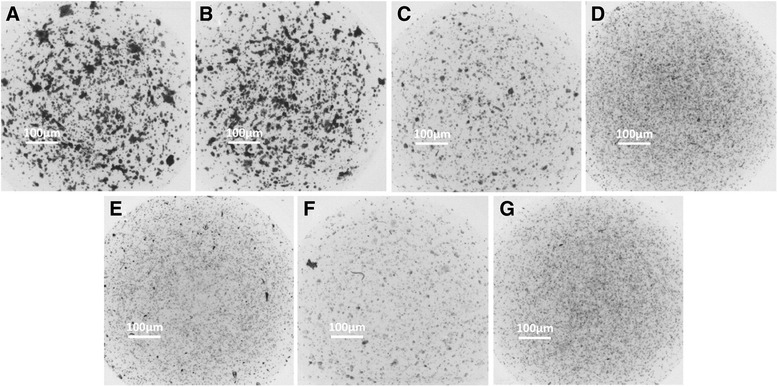
The represent figures of *IC*_*max*_ concentration anti-tumor agents on 3-D cultured HepG2 cells. **a** HepG2 cells were cultured in a 3-D manner. **b-g** HepG2 cells were treated with solvent control (**b**), or ICmax concentration of agents, 2.0 μg/ml 5-FU (**c**), 0.08 μg/ml PAC (**d**), 2.0 μg/ml CDDP (**e**), 0.5 μg/ml EPI (**f**) or 5.0 μg/ml L-OHP (**g**)

Next, the effect of anti-tumor agents on HepG2 was detected by CD-DST (Fig. 2). The results showed that HepG2 was sensitive to 5-PU, PAC, CDDP, EPI, or L-OHP, and the *IC*_*50*_ values were 0.83 ± 0.45 μg/ml, 0.03 ± 0.02 μg/ml, 1.15 ± 0.75 μg/ml, 0.09 ± 0.03 μg/ml, and 1.76 ± 0.44 μg/ml, respectively. Moreover, the *IC*_*max*_ values were 2.0 μg/ml, 0.08 μg/ml, 2.0 μg/ml, 0.5 μg/ml, and 5.0 μg/ml, respectively. The representative photographs are shown in Figs. 3 and 4.

### Sensitivity of primary cells separated from HCC clinical specimens

**Fig. 5:**
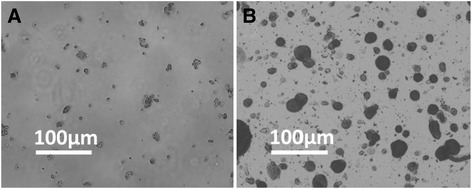
Primary cells separated from clinical specimens culture in collagen gel droplets. Primary cells separated from clinical specimens were seeded in collagen gel droplets and analyzed by a multifunctional microplate reader at 540 nm. **a** The represent grey scale photograph of baseline (0-time) cells; (**b**) The represent grey scale photograph of cells after 5–7 days culturing

**Fig. 6:**
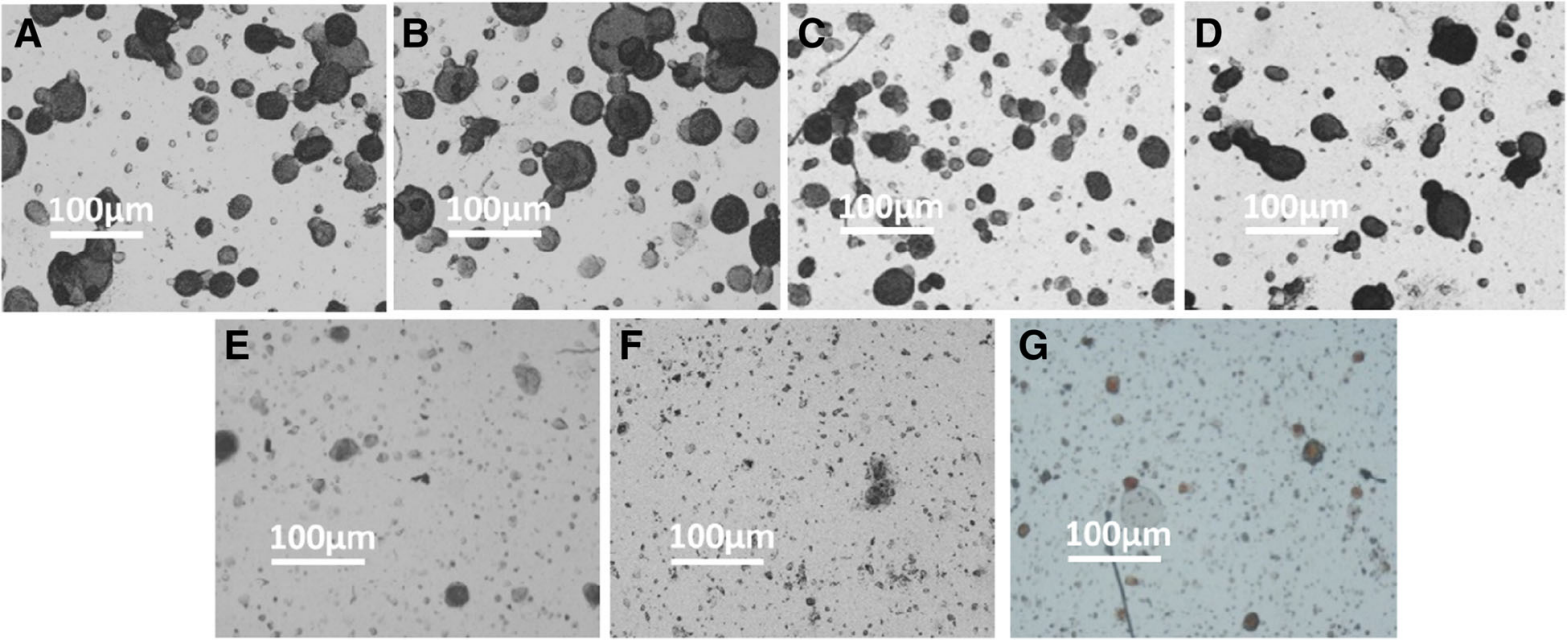
The represent figures of patients are sensitive to *IC*_*max*_ concentration anti-tumor agents. **a** Primary cells separated from clinical specimens were cultured in a 3-D manner. **b-g** Primary HCC cells were treated with solvent control (**b**), or ICmax concentration of agents, 2.0 μg/ml 5-FU (**c**), 0.08 μg/ml PAC (**d**), 2.0 μg/ml CDDP (**e**), 0.5 μg/ml EPI (**f**) or 5.0 μg/ml L-OHP (**g**)

Next, the sensitivity of primary cells separated from HCC clinical specimens to anti-tumor agents was detected by CD-DST methods (Fig. 5). Cells were treated with an *IC*_*max*_ or *IC*_*50*_ concentration of antitumor agents. As shown in Table 4, 8/26, 9/26, and 5/26 patients may be sensitive to *IC*_*max*_ values of CDDP, EPI, or L-OHP, and only 3/26, 4/26, and 2/26 patients may be sensitive to *IC*_*50*_. However, no patients were sensitive to 5-Fu or PAC. The represent photographs are shown in Fig. 6. The characteristics of the tumor (histological grade, BCLC grade number of nodules) in relation to the sensitivity to chemotherapeutic agents are shown in Table 5. Taken together, patients suffering from HCC were not sensitive to PAC or 5-Fu, and only a few obtained clinical benefits from CDDP, EPI, or L-OHP.

**Table 4:** the sensitivity of patients to anti-tumor agents

Compounds	Total patients	Patients were sensitive to compounds	
		*IC*_*max*_ group	*IC*_*50*_ group
5-FU	26	–	–
PAC	26	–	–
CDDP	26	8	3
EPI	26	9	4
L-OHP	26	5	2

**Table 5:** characteristics of the tumor in relation to sensitivity to chemotherapeutic agents

Compounds		Sensitive patients	BCLC		Child-Pugh		Tumor Number		Tumor differentiation		
			A	B	A	B	Single	2~3	Well	moderate	poorly
CDDP	*IC*_*max*_	8	6	2	8	–	5	3	7	1	–
	*IC*_*50*_	3	3	–	3	–	2	1	3	–	–
EPI	*IC*_*max*_	9	5	4	9	–	6	3	4	5	–
	*IC*_*50*_	4	3	1	4	–	2	2	2	2	–
L-OHP	*IC*_*max*_	5	5	–	5	–	3	2	5	1	–
	*IC*_*50*_	2	2	–	2	–	1	1	2	–	–
5-FU	*IC*_*max*_	–	–	–	–	–	–	–	–	–	–
	*IC*_*50*_	–	–	–	–	–	–	–	–	–	–
PAC	*IC*_*max*_	–	–	–	–	–	–	–	–	–	–
	*IC*_*50*_	–	–	–	–	–	–	–	–	–	–

### 2-D cultured HepG2 cells were more sensitive to anti-tumor agents

Moreover, to confirm the sensitivity of HCC cells to anti-tumor agents, the effect of agents on HepG2 or HepG2/ADR, an MDR cell line, was examined by MTT assays and the *IC*_*50*_/*IC*_*max*_ concentrations were calculated. The results are shown in Table 6. The *IC*_*50*_ value of agents on HpeG2 cells obtained from the MTT assays were much lower than those obtained from CD-DST. Moreover, the *IC*_*50*_ value of agents on HpeG2/ADR cells obtained from the MTT assays were similar with those obtained from CD-DST. These results indicated that sensitivity of 2-D cultured HepG2 cells to anti-tumor agents was much higher than that in 3-D-cultured HepG2 cells.

**Table 6:** the sensitivity of HepG2 or HepG2/ADR cells to antitumor agents

Compounds	2-D HepG2	2-D HepG2/ADR	3-D HepG2
	*IC*_*50*_ values (μg/ml)		
5-PU	0.45 ± 0.06	0.77 ± 0.33	0.83 ± 0.45
PAC	0.01 ± 0.00	0.02 ± 0.01	0.03 ± 0.02
CDDP	0.26 ± 0.23	1.39 ± 0.40	1.15 ± 0.75
EPI	0.02 ± 0.01	0.16 ± 0.04	0.09 ± 0.03
L-OHP	0.55 ± 0.12	1.85 ± 0.44	1.76 ± 0.44

## Discussion

HCC is currently one of the most common malignancies, especially in China and the Asia-Pacific region. Although an oral multi-targeted kinase inhibitor, sorafenib, has been used as a curative approach, cytotoxicity chemotherapeutic agents are still treatment choices, e.g.*,* oral medication or hepatic artery chemotherapy and transcatheter arterial chemoembolization (TACE) [21–25]. 5-FU, CDDP, EPI, and L-OHP can induce double DNA strand breaking (DSB) and block DNA replication via multi-mechanisms [26]. PAC targets microtubules and disrupts cell division [27]. It is well known that HCC may be resistant to almost all kinds of cytotoxicity chemotherapeutic agents due to its multi-drug resistance (MDR) features. During the MDR process, breast cancer resistance protein (BCRP), multi-drug resistance relative protein2/3 (MRP2/3) or multi-drug resistance 1 (MDR-1) may participate in the un-anticipated efficacy loss of chemotherapeutic agents via the phase I or phase II drug metabolizing process [28]. Some other signaling pathways, e.g. JAK/STAT (janus kinase / signal transducers and activators of transcription), PI3K/mTOR (phosphatidylinositol 3-kinase / mammalian target of rapamycin) or hypoxia-related pathways, would also participate in the MDR of HCC [29, 30].

Moreover, a higher level of these resistance genes has been confirmed in HCC/liver clinical specimens than that in HCC cell lines. Tumor cells in solid tumors grow in a 3-D pattern. Therefore, it is urgent to establish an in vitro model reflecting the 3-D culture of tumor cells. To date, there is no effective or rapid testing system for screening sensitive drugs for HCC patients to guide the clinical individual chemotherapy. For the first time, our presence work establishes the application of CD-DST in HCC chemotherapeutic-efficacy examination. The sensitivity of HepG2 or primary cells separated from HCC clinical specimens to 5-FU, PAC, CDDP, EPI, and L-OHP was identified. No patients were sensitive to PAC or 5-FU. As shown in Table 5, the characteristics of the tumors (histological grade, BCLC grade number of nodules) in relation to their sensitivity to chemotherapeutic agents were identified. Patients with BCLC A or well-differentiated HCC would more sensitive to CDDP or L-OHP, whereas patients with BCLC B or moderate-differentiation HCC would be more sensitive to EPI than CDDP or L-OHP. Moreover, patients with poor-differentiation HCC would not be sensitive to anti-tumor agents, and the tumor numbers seemed unrelated to anti-tumor agent sensitivity.

Traditionally, the biological behavior of cancer cells was determined using 2-D methods, e.g., MTT or colony formation. However, 2-D methods may be insufficient to reflect the in vivo behavior of cancer cells in solid tumor tissues [7]. Thus, it is valuable to develop and validate a rapid method to examine the biological behavior of cancer cells in a 3-D manner. Tissue engineering research, e.g., 3-D spatial array, investigated the involvement of the extracellular matrix (ECM) in solid tissues [7]. The interaction between cells and the ECM modulates the behavior and features of solid tumors [8–10]. Collagen is a main constituent of the ECM and supports the formation of the microenvironment in solid tumors [8–12]. CD-DST is a mature test in which primary-culture cells of HCC are separated from surgical resection specimens embedded in collagen droplets [7–12]. Applications of CD-DST in chemotherapeutic sensitivity examination have been demonstrated in some kinds of human cancers, e.g., lung cancer, gastric cancer, breast cancer, and colorectal cancer [8–12]. Higashiyama et al. (2010) predicted the chemotherapeutic effect on postoperative recurrence in non-small cell lung cancer patients [9]. Sun et al. (2013) established a 3-D gastric tumor culture model [10]. Lin et al. (2015) revealed the effect of pyruvate kinase M2 on Epirubicin and 5-Fluorouracil in breast cancer by CD-DST [11]. Takahashi et al. (2015) examined the anti-tumor activity of fucoxanthin and fucoxanthinol on cell lines or primary cells from colorectal cancer clinical specimens [12]. In the present work, for the first time, we developed and validated an effective method to predict the resistance of HCC patients to anti-tumor agents. The result showed that 2-D cultured HepG2 cells were more sensitive to anti-tumor agents than cells cultured in a 3-D manner but similar to a 2-D cultured resistance cell line, HepG2/ADR. Moreover, primary cells separated from clinical specimens were much more resistant to anti-tumor agents. Therefore, CT-DST, a 3-D tumor model, is valuable for evaluating and mimicking the in vivo efficacy of antitumor agents.

## Conclusions

The in vitro drug sensitivity exanimation validated the MDR features of HCC. CD-DST may be a useful method to predict the potential clinical benefits of anticancer agents for HCC patients.

## Abbreviations

5-FU: 5-furuolouracil
BCRP: Breast cancer resistance protein
CDDP: Cisplatin
CD-DST: Collagen gel droplet embedded 3D–culture system
EPI: Epirubicin
HCC: Hepatocellular carcinoma
IR: Inhibitory rate
JAK: Janus kinase
L-OHP: Oxaliplatin
MDR: Multi-drug resistance
MDR-1: Multi-drug resistance 1
MRP2/3: Multi-drugs resistance relative protein2/3
mTOR: Mammalian target of rapamycin
PAC: Paclitaxel
PI3K: Phosphatidylinositol 3-kinase
STAT: Signal transducers and activators of transcription
TACE: Transcatheter arterial chemoembolization
